# The Voltage-Dependent Deactivation of the KvAP Channel Involves the Breakage of Its S4 Helix

**DOI:** 10.3389/fmolb.2020.00162

**Published:** 2020-07-29

**Authors:** Olivier Bignucolo, Simon Bernèche

**Affiliations:** ^1^Biozentrum, University of Basel, Basel, Switzerland; ^2^SIB Swiss Institute of Bioinformatics, Basel/Lausanne, Switzerland

**Keywords:** Kv channel, resting state, molecular dynamics, voltage-sensor domain, pore domain, avidin accessibility

## Abstract

Voltage-gated potassium channels (Kv) allow ion permeation upon changes of the membrane electrostatic potential (Vm). Each subunit of these tetrameric channels is composed of six transmembrane helices, of which the anti-parallel helix bundle S1-S4 constitutes the voltage-sensor domain (VSD) and S5-S6 forms the pore domain. Here, using 82 molecular dynamics (MD) simulations involving 266 replicated VSDs, we report novel responses of the archaebacterial potassium channel KvAP to membrane polarization. We show that the S4 α-helix, which is straight in the experimental crystal structure solved under depolarized conditions (Vm ∼ 0), breaks into two segments when the cell membrane is hyperpolarized (Vm << 0), and reversibly forms a single straight helix following depolarization (Vm = 0). The outermost segment of S4 translates along the normal to the membrane, bringing new perspective to previously paradoxical accessibility experiments that were initially thought to imply the displacement of the whole VSD across the membrane. The novel model is applied through steered and unbiased MD simulations to the recently solved whole structure of KvAP. The simulations show that the resting state involves a re-orientation of the S5 α-helix by ∼ 5–6 degrees in respect to the normal of the bilayer, which could result in the constriction and closure of the selectivity filter. Our findings support the idea that the breakage of S4 under (hyper)polarization is a general feature of Kv channels with a non-swapped topology.

## Introduction

Voltage-gated potassium channels (Kv) are tetramers that open and close as a function of the membrane electrostatic potential (Swartz, 2008). Each subunit is composed of six transmembrane helices S1–S6. Voltage dependence is granted by helices S1 to S4, an anti-parallel helical bundle constituting the voltage-sensor domain (VSD), which is linked to the pore domain composed of helices S5 and S6. A much-conserved structural feature of the voltage-sensor domains is a series of four to six basic residues distributed along the S4 helix, each one followed by two hydrophobic residues. The voltage sensing properties are attributed to these positively charged residues, which are assumed to respond to the membrane electrostatic potential (Vm) by a translation along the membrane normal. This results in an apparent charge transport, or gating current (Tempel et al., 1987; Bezanilla, 2008; Li et al., 2014). Under depolarized potential, the pore is open and the channel enters its active state, which can be determined experimentally. The resting or closed state under polarized potentials has been more challenging to investigate.

A “consensus” mechanism describing the voltage-dependent conformational changes of Kv channels in response to variations of the membrane electrostatic potential was developed (Vargas et al., 2012) through the integration of several computational studies, notably based on the structures of the Kv1.2 and Kv1.2/2.1 chimera channels (Yarov-Yarovoy et al., 2006; Nishizawa and Nishizawa, 2008, 2009; Denning et al., 2009; Treptow et al., 2009; Delemotte et al., 2010, 2011; Schwaiger et al., 2011; Vargas et al., 2011; Domene, 2012). This helical screw model consists of a sliding helix mechanism in which S4 undergoes an ensemble of transitions toward the resting state, involving a rotation and a translation along its main axis and toward the intracellular compartment. The translation is ∼ 10 Å long, with a spread of 3–4 Å. While most of the current knowledge on eukaryotic Kv channels was incorporated in this model, data from the archaea KvAP channel were not included, suggesting that the KvAP structure was incompatible with the proposed mechanism.

**FIGURE 1:**
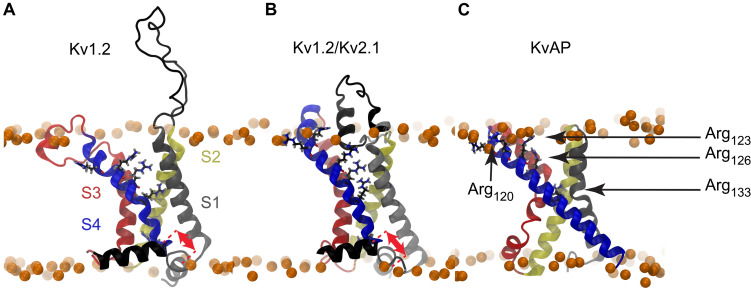
Molecular representations of Kv channel structures. The crystal structures of **(A)** Kv1.2, **(B)** Kv1.2/Kv2.1, and **(C)** KvAP are shown embedded in a lipid bilayer (Phosphorus atoms shown as orange spheres). The red arrows illustrate the different translational motions that S4 is likely to undergo without exposing any hydrophobic residue outside of the bilayer core. Such a translation seems impossible in the case of the KvAP crystal structure, thus no arrow is drawn in panel **C**. The helices are colored as follows: S1: gray, S2: yellow, S3: red, S4: blue. The arginine residues of the S4 N-ter are shown as sticks with carbon, nitrogen, oxygen in black, blue, and red.

The structure of the KvAP voltage sensing domain from the Archaea Aeropyrum Pernix was solved by crystallography and NMR (Jiang et al., 2003a; Butterwick and MacKinnon, 2010; Shenkarev et al., 2010), and more recently its complete structure was solved by cryo-EM (Tao and MacKinnon, 2019). Since these experiments were performed in the absence of any membrane voltage, only the active or inactivated state of the KvAP VSD could be captured. Some features of its elusive resting state have nevertheless been characterized by several experimental biophysical studies (Jiang et al., 2003b; Cuello et al., 2004; Ruta et al., 2005; Biverstahl et al., 2009). It is noteworthy that the sliding helix model depends on the possibility of S4 to exert a translation along its axis in response to variation of the membrane electrostatic potential. As shown in Figures 1A–C, while such a movement is plausible for Kv1.2 and Kv1.2/Kv2.1, there is essentially no room for the long S4 helix of KvAP (33 vs 20 residues) to slide upon depolarization without exposing hydrophobic residues to the polar environment of phospholipid head groups or the solvent.

The mechanistic interpretation of accessibility measurements of avidin to biotinylated cysteines in the S3 and S4 helices of KvAP remained elusive since their publication (Jiang et al., 2003b; Ruta et al., 2005). These experiments showed that avidin in the intracellular space could bind to biotinylated cysteine located in the middle of S4. This observation was explained by a large displacement of the whole S4 helix across the hydrophobic core of the membrane. However this large displacement was difficult to reconcile with other experiments (Ahern, 2004).

Using molecular dynamics simulations, we investigated on the possible conformational changes induced by membrane polarization on the isolated voltage-sensing domain of KvAP (*n* = 130 replications of the VSD). Under hyperpolarized conditions, a reversible conformational change that involves the breakage of helix S4 was observed. The resulting sliding movement of the N-ter segment of S4 along the normal to the membrane is further shown, using steered molecular dynamics (SMD) simulations, to provide a coherent explanation to the avidin accessibility measurements by the MacKinnon lab (Jiang et al., 2003b; Ruta et al., 2005). Following the breakage of S4, its C-ter segment reorient itself along the lipid head groups, in a conformation reminiscent of the S4-S5 linker in eukaryotic Kv channels. We then asked what is the impact of the reorientation and displacement of the C-ter segment on the pore domain. The conformational changes observed on the isolated VSD were transferred to the whole KvAP channel using SMD simulations, and the systems were then further submitted to a range of transmembrane voltages. We observed a correlation between the conformation of helix S4 and the orientation of both helix S5 and the pore helix. Based on these simulations, we propose that under membrane polarization, the breakage of S4 allows for a displacement of its C-ter segment in the plane of the membrane that allosterically leads to the constriction of the channel structure around the selectivity filter, possibly blocking ion permeation.

## Materials and Methods

### Molecular Dynamics Simulations of the Isolated VSD

The atomic model of the KvAP VSD was based on the crystal structure PDB code 1ORS, assumed to correspond to the active state of the channel (Berman et al., 2000). The VSD was inserted in an asymmetric bilayer using the CHARMM-GUI web service (Jo et al., 2009). The “extra-cellular” leaflet was composed of 100 POPC (1-palmitoyl-2-oleoyl-sn-glycero-3-phosphocholine) and 80 cholesterol, and the “intra-cellular” leaflet was composed of 50 POPC, 50 POPS (1-palmitoyl-2-oleoyl-sn-glycero-3-phosphoserine) and 80 cholesterol molecules. The system was further solvated with ∼ 25,000 water molecules, represented by the TIP3P model (Jorgensen et al., 1983). Neutralizing K^+^ and Cl^–^ counterions were added to mimic a salt concentration of 0.15 M. Two such systems were combined in an antiparallel way to form a double bilayer system, simulating a cell membrane separating two different water compartments (Demchenko and Yesylevskyy, 2009; Gurtovenko and Vattulainen, 2009; Kutzner et al., 2011). The system contained ∼ 235,000 atoms. The construct contains in its center a water slab simulating the “intracellular” compartment, and the two slabs on the edges are combined through periodic boundary conditions to form the “extracellular” compartment. Using this construct, the membrane potential can be adjusted to the desired value by changing the number of ions in either compartment. Consequently, the system described above was replicated 65 times, in order to generate an ensemble of conditions with simulated membrane potential (Vm) ranging from −1.7 to 0.5 V. These high values allowed for the exploration of a larger conformational space, however without attaining potentials that would induce electroporation. In a study investigating the stabilization effect of cholesterol on lipid membranes, Casciola et al. (2014) exposed bilayers composed of 0 to 50 mol% cholesterol to membrane potential values up to 5.35 V. They observed within the first 60–70 ns of simulation that the “electroporation thresholds increased from ∼ 2.3 V for bare bilayers to ∼ 4.4 V as the cholesterol content reached 30 mol% concentration”. In another study, an electroporation thresholds of −1.8 V was reported for a cholesterol free membrane (Polak et al., 2013). According to these data, the membrane potential applied in our work is not expected to destabilize the bilayers, which contain ∼ 45 mol% cholesterol. We effectively did not observe any strong membrane deformation during the simulations, generally of ∼ 200 ns length, reaching 740 ns in one case.

All-atom MD simulations were performed with the GROMACS software package version 4.5 (Van Der Spoel et al., 2005), with the CHARMM force-field (MacKerell et al., 1998), versions v27 for proteins (Mackerell et al., 2004) and v36 for lipids (Klauda et al., 2010). A constant pressure of 1 bar was maintained using the Berendsen algorithm (time constant 1ps) (Berendsen et al., 1984). The temperature was kept at 310 K by a stochastic rescaling of the velocities (time constant 0.2 ps) (Bussi et al., 2007). Bond lengths and angles involving hydrogen atoms were constrained using the LINCS algorithm (Hess et al., 1997), allowing an integration time step of 2 fs. Short-range electrostatics were cut off at 1.2 nm, and the particle mesh Ewald method was used for long-range electrostatic (Essmann et al., 1995). Van der Waals interactions were described with Lennard-Jones potential up to a distance of 1.2 nm. The systems were equilibrated following the CHARMM-GUI protocol (Jo et al., 2007). Independent simulations were conducted on 65 double bilayer systems, thus allowing the study of 130 voltage-sensor domains at various membrane potentials.

### Avidin Accessibility Simulations

For the study of the avidin accessibility, we reasoned that whereas avidin generally forms a tetramer in solution, with extremely high affinity to biotin (K_d_ ∼ 10^–15^ M), it was shown that the monomeric avidin also binds biotin with high affinity (K_d_ ∼ 10^–7^ M), which is sufficient to explain the experiments mentioned in the main text (Regnier and Cho, 2013). The access of a monomeric avidin to a VSD-bound biotin is structurally less constrained than that of a tetramer. Consequently, chain A from the complex avidin-biotin crystal structure (PDB code 1AVD) was placed at a few angstroms from a bilayer containing KvAP, which was in either the crystallized conformation or after breakage of S4. Force field parameters for biotin were generated using the CGenFF web-service (Vanommeslaeghe et al., 2010) based on a structure obtained from the Zinc12 database (Irwin et al., 2012). In the avidin accessibility work by Jiang et al. (2003b), residues Leu125 and Ile127 are the only residues found at the level of the extracellular leaflet of the membrane in the X-ray structure that became also accessible from the intracellular side upon membrane polarization. For our investigation, we selected Ile127. A constant pulling force of 100 kJ⋅mol^–1^⋅nm^–1^ was applied between the Ca atom of Ile_127_ of KvAP and the center of mass of the biotin carboxylic acid group. An equal force was applied between the COM of the nitrogen and Sulfur atoms of biotin and that of Trp_70_ and Trp_97_ of avidin, as these two residues define the biotin-avidin binding site (Livnah et al., 1993).

### Molecular Dynamics Simulations of the Whole KvAP Channel

The recently characterized structure of the entire KvAP channel, PDB code 6UWM (Tao and MacKinnon, 2019), was inserted in a bilayer containing ∼310 POPC molecules. The system was further hydrated with ∼ 53,000 water molecules at 150 mM KCl. Since the purpose of this study was to investigate how the kink in S4 might affect the conformation of the pore domain, the knowledge acquired from the VSD simulations was directly applied to this setting. In order to facilitate the rupture of the Asp_62_-Arg_133_ salt bridge, Asp_62_ was protonated. A short steered molecular dynamics simulation was conducted, in which the Cα atoms repelled each other by a force of 23 kJ⋅mol^–1^⋅nm^–1^ and the Cα atoms of G_134_ were pulled toward the intracellular space by a force of 120 kJ⋅mol^–1^⋅nm^–1^. The simulation was stopped after at least two of the Asp_62_-Arg_133_ salt bridges were broken, which occurred after 23 ns.

The obtained structure was then used to construct 17 double bilayer systems. Since each system contained two tetrameric channels, thus 8 VSDs, a total of 136 VSDs were investigated. These systems were then exposed for another 200 to 250 ns to membrane potentials ranging from −0.79 to 0.01 Volts. Asp_62_ was kept protonated during these unbiased simulations to reduce the risk that S4 would retrieve its straight conformation before structural changes could be transmitted to the pore domain. The duplication of the molecular system during the generation of the double bilayer, which is then followed by the replication into several molecular systems, bears the risk of introducing biases in the statistical analyses (Hurlbert, 1984). To assure of the independence of the individual systems and to consider only trajectory data that were properly randomized, we performed the following test. The orientation of the S4 C-ter with respect to the normal of the bilayer was computed and averaged for each subunit in each simulation over the full length of the trajectory, starting at different time points: *t* = 10, 25, 50, 75, 100, 125, 175, 180, and 200 ns. A single-way ANOVA of the S4 C-ter orientation as variable and the identity of the subunits of the tetrameric protein (e.g., A, B, C, and D) as fixed factor was performed for each set. The *p* values as a function of the initial time point are shown in Supplementary Figure S2. It shows that the orientation of the S4 C-ter is significantly correlated to the identifier of the subunit when only 10, 25, or 50 ns are discarded. They become rapidly decorrelated (α threshold 0.05) if more than 50 ns are discarded, but the *p*-values reach a plateau. After 150 ns the *p*-values increase dramatically, suggesting that the trajectories become rather independent from the initial conditions. To have some margin, 180 ns of trajectory were discarded for the statistical analyses.

The all-atom simulation parameters were similar to those used for the isolated VSD (see above). The orientation of the helices was determined as follows. The protein structures of all frames were aligned to the initial structure, initially oriented with its principal axis along the *z*-axis and orthogonal to the bilayer. Each α-helix was represented by a vector. The starting and ending points of the vector were the centers-of-mass (COM) of the backbone atoms of residues 25–28 and 42–45 for S1, 52–55 and 72–75 for S2, 115–118 and 129–132 for S4 N-ter, 134–137 and 143–146 for S4 C-ter, 150–153 and 167–170 for S5, 207–210 and 235–238 for S6, and 182–185 and 192–195 for the pore helix. For each frame, the cosine of this vector in respect to the normal of the bilayer was then calculated.

### Sequence Analysis

The non-redundant UniProt/SwissProt sequence database was used for retrieving voltage-gated potassium channels homologs to the KvAP VSD sequence, ID Q9YDF8 (Altschul et al., 1997; SIB Swiss Institute of Bioinformatics Members, 2016). The sequences were further curated using in-house Python scripts in order to remove undefined species and uncharacterized fragments, and to retain sequences of length similar to the KvAP VSD ± 100 residues. The scripts further selected sequences characterized by the typical feature of a voltage-sensor domain, i.e., a series of three triplets consisting of a pair of mostly hydrophobic residues followed by a basic residue and, in addition, a segment of seven any residues followed by a Gly. This last criterion allowed us to discriminate the sequences according to the specificity of the S4 helix described in this work. The alignment was generated in Chimera (Pettersen et al., 2004), using the Clustal Omega algorithm (Sievers et al., 2011). For completeness, an alignment of KvAP and HCN1 was added in the sequence alignment.

### Data Analysis

Data were analyzed with R, python, VMD, tcl in-house scripts and in Prism (Graphpad Prism 8). Details are provided in the results section and figure legends, where appropriate. In cases of multiple comparisons, the Šidák’s multiple comparison test was used to adjust the probability values.

**FIGURE 2:**
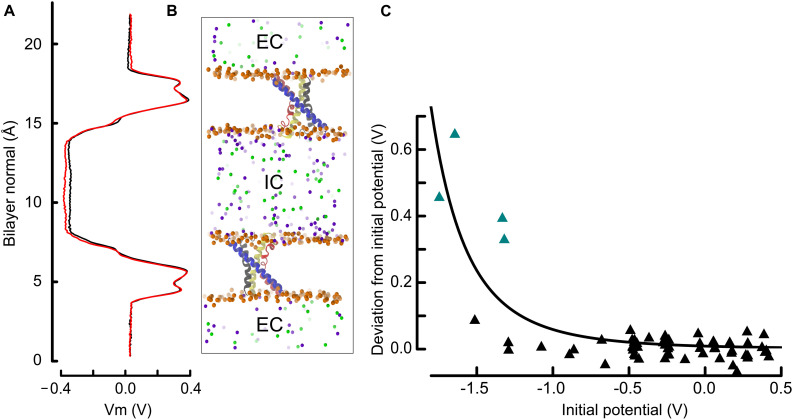
The double bilayer system enables the explicit tuning of the membrane potential and facilitates the identification of gating charge transport events. **(A)** The electrostatic potential along the bilayer normal (z) is shown adjacent to the molecular representation. We draw the potential at the beginning (0–20 ns, black) and at the end (last 20 ns, red), which is normalized to 0 V at *z* = 0, i.e., in the EC. The similarity of the two curves indicates that the charge imbalance manually set between the two compartments remained stable during the simulation. **(B)** Molecular representation of two antiparallel bilayers with embedded VSDs. The proteins are represented and colored as in Figure 1 and the phosphorous atoms as well as the ions are depicted as spheres. Phosphorous, sodium and chloride are colored orange, violet, and green. For clarity, water and lipid molecules have been removed. All shown atoms constitute one simulated entity. Because of the periodic boundary conditions, the water and ions above and below each displayed bilayer communicate and form a single compartment (EC: extracellular), which is isolated from the middle slab (IC: intracellular). **(C)** Deviation from the initial membrane potential at *t* = 180 to 200 ns as a function of the initial potential. The four simulations identified in this study, and in which a charge transport occurred are highlighted as teal triangles. The continuous line corresponds to a least square fit power function of the initial potential (see section “Materials and Methods”). The observed and fitted deviations from the initial potential are correlated with *R* = 0.82, and p < 0.001 for linear regression.

To ensure that the deviations from the initial membrane potential (*t* = 0 ns) of a given simulation, hereafter named Vm_0_, were correlated with Vm_0_, the data were fitted (least squares) with a power function (continuous line in Figure 2C) of the form:

$$f\,\left(V\,m_{0}\right)=\frac{c}{{\left(V\,m_{0}+k\right)}^{h}}$$

where the parameter c caps the deviation value and h is a slope factor. The function Vm_0_ + k controls the position of the denominator along the *x* axis. For the data presented in Figure 2C, the parameter c was set arbitrarily to 0.7 and the values of 4.24 and 2.79 were found for h and k. Following this fit, a simple-way ANOVA could be computed between the observed and calculated deviations from the initial potential Vm_0_, which hold p < 0.001 and a coefficient of correlation of 0.82, supporting the idea that the strong deviations from the initial potential occur under high depolarization.

## Results

### KvAP Response to Membrane Polarization Involves Breakage of S4

To address the ill-defined mechanism of voltage-sensing in prokaryotic cells, an ensemble of 65 independent molecular dynamics (MD) simulations were performed, exposing 130 voltage-sensor domains (VSDs) to a wide range of membrane electrostatic potentials. As shown in Figure 2, the simulation system consisted of two bilayers mimicking a cell with two separated water compartments that, according to the orientation of the bilayer leaflets and incorporated proteins, correspond to the extra- and intracellular compartments. This compartmentalization allows one to adjust the membrane potential by changing the number of ions in either compartment (Gurtovenko, 2005; Denning and Woolf, 2008; Gurtovenko and Vattulainen, 2009). Thus, each individual simulation, of length ∼ 200 ns on average, allowed the investigation of two VSDs. The simulated membrane potential (Vm) ranged from −1.7 to 0.5 V (see Figure 2C). While these Vm values are of higher magnitude than physiologically found in cells, they are close to values used in previous works and remain in a range that does not expose the membrane to electroporation (see details in section “Materials and Methods”).

We controlled the electrostatic steadiness of the bilayer systems by monitoring the difference ΔVm between the membrane potential averaged over the first and last 20 ns of the trajectory (see Figure 2). As a consequence of the limited size of the systems, which typically contain ∼ 240,000 atoms, a single charge transport across the membrane induces a ΔVm of ∼ 200 mV. In four simulations performed under hyperpolarized voltages, we detected variations of the membrane potential that correspond to the relocation of two charges across the membrane (teal triangles in Figure 2C). There is a highly significant (p < 0.001) negative correlation between the charge transport occurrence and the membrane potential, indicating that these events are most likely related.

**FIGURE 3:**
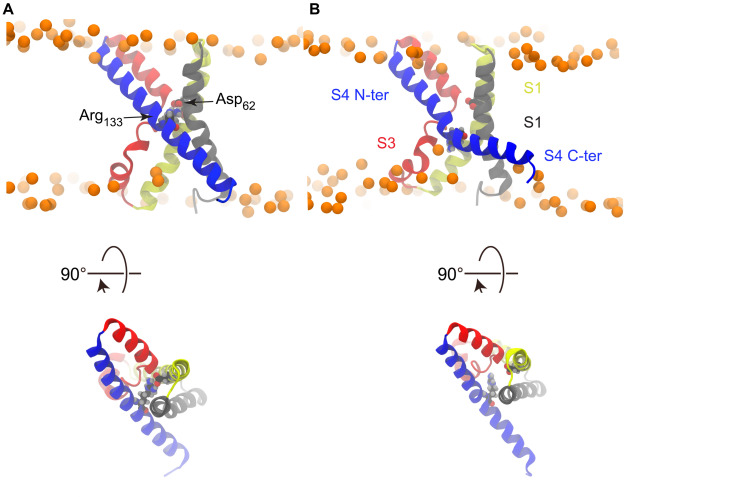
Salt bridge rupture upon polarization. **(A)** S4 is straight and the Asp_62_-Arg_133_ salt bridge is formed in the X-ray structure. **(B)** Representative snapshot from a simulation conducted under hyperpolarized potential, taken after the charge transport event, showing the disrupted salt bridge and broken S4.

**FIGURE 4:**
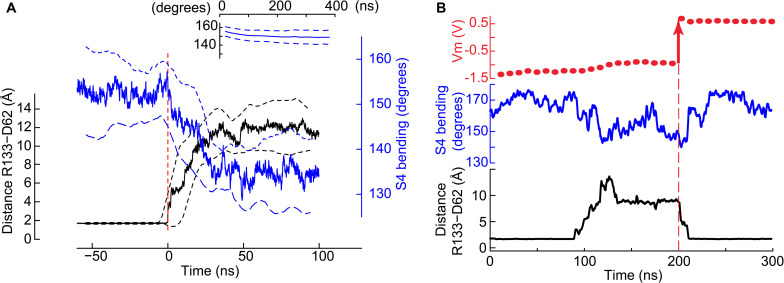
Co-occurrence of the salt bridge rupture and the S4 breakage upon hyperpolarization. **(A)** Time series of the minimal distance between Asp_62_ and Arg_133_ (black, left *y*-axis) and the bending of S4 (blue, right *y*-axis). Mean ± SD from four pooled independent trajectories are shown. Before pooling, the time axis of each trajectory was set to 0 when the Asp_62_ – Arg_133_ distance goes above 3 Å for the first time (red dashed vertical line). For comparison, the inset shows the S4 bending angle time evolution (mean ± SD) of trajectories in which the distance between Asp_62_ and Arg_133_ remained stable at ∼ 2Å (n_VSD_ = 126). **(B)** The status of the Asp_62_-Arg_133_ salt bridge and the bending of helix S4 depend upon the membrane potential. Shown are the membrane potential (above), the bending of S4 (middle) and the minimum distance between Arg_133_ and Asp_62_ as a function of the simulation time. At t∼90 ns, rupture of the salt bridge. At t∼210 ns, restoration of the salt bridge. The vertical black dashed line highlights the time point at 200 ns at which the simulation was stopped, ions were displaced to generate a positive membrane potential (red arrow at Vm) and the simulation restarted.

The crystal structure of KvAP is characterized by two cavities readily accessible to the solvent, as can be seen in Figure 1C. As shown in previous works (Sands et al., 2006; Sands and Sansom, 2007), whereas water can insert deeply toward the middle of the membrane from the extra- and intracellular sides, interacting with several polar residues, its permeation is prevented by the salt bridge linking Asp_62_ (helix S1) and Arg_133_ (helix S4) (Figure 3). This salt bridge thus constitutes the unique barrier between the extra- and intracellular compartments. We observed the rupture of this salt bridge in all simulations in which a charge transport occurred (Figures 3, 4), and in none of the others. Upon rupture of the salt bridge, the side-chains reoriented so that the negative charge of Asp_62_ moved toward the extracellular compartment, while the positive charge of Arg_133_ moved toward the intracellular compartment. This resulted in the observed gating charge transport. In addition, whereas the S4 helix was straight under depolarized conditions, it broke at the level of Gly_134_ only when the membrane was hyperpolarized and the salt bridge was broken as well. Consequently, S4 was split in two segments, the one on the intracellular side being reoriented parallel to the membrane surface (Figure 3B), like the S4-S5 linker of Kv1.2 and Kv1.2/Kv2.1 (Figures 1A,B).

A time series analysis of the four independent trajectories in which the gating charge transport took place shows that in each of them the rupture of the salt bridge and of S4 occurred concurrently (Figure 4A). None of these events was observed in the simulations conducted at Vm ∼ 0 V (Figure 4A, inset). This time series analysis supports the conclusions that 1) the two conformational changes are related and 2) they are due to the membrane polarization. We further asked whether the membrane potential suffices to control the status of the Asp_62_-Arg_133_ salt bridge. In a particular simulation initialized under a hyperpolarizing potential, the salt bridge broke after ∼ 90 ns. Consecutively to the reorientation of the Asp_62_ and Arg_133_ side chains, the membrane potential decreased within ∼ 30 ns to a value corresponding to a gating charge transport of two units and remained stable during the next 50–60 ns. With the aim of testing the dependence of the conformational changes on the membrane potential, we stopped the simulation and moved ions between the extra- and intracellular compartments in order to reach a potential of ∼+0.5 V. We then continued the simulation from this new starting point. Within ∼10 ns of simulation, the side chains of Asp_62_ and Arg_133_ reoriented and restored the salt bridge, which remained intact for the next 200 ns, of which the first 100 ns are shown in Figure 4B. This figure also shows that the bending of S4 correlates with the state of the salt bridge, with a tendency to become straight again at Vm ∼ 0.5 V.

### Elucidation of Experiments Involving Avidin Binding to Biotinylated KvAP Voltage-Sensor Domain

In 2003 and 2005, Jiang et al. (2003b) and Ruta et al. (2005) reported experiments in which the binding of avidin to biotinylated cysteines was used to deduce the residue accessibility from the external or internal cell compartments. In the study described by Jiang et al., a 17Å linker connected the Cys Cα atom to biotin through an amide bond. These avidin binding experiments notably showed that the biotinylated residues 125 and 127, located in the upper half of the S4 helix, were accessible to avidin from the intracellular compartment. These results supported the idea of a voltage-sensor paddle model in which the helix-turn-helix S3a-S4 moves through the membrane upon voltage changes. However, this model requires an important movement of charged amino acids across the hydrophobic core of the membrane, which was difficult to reconcile with other observations (Ahern, 2004).

**FIGURE 5:**
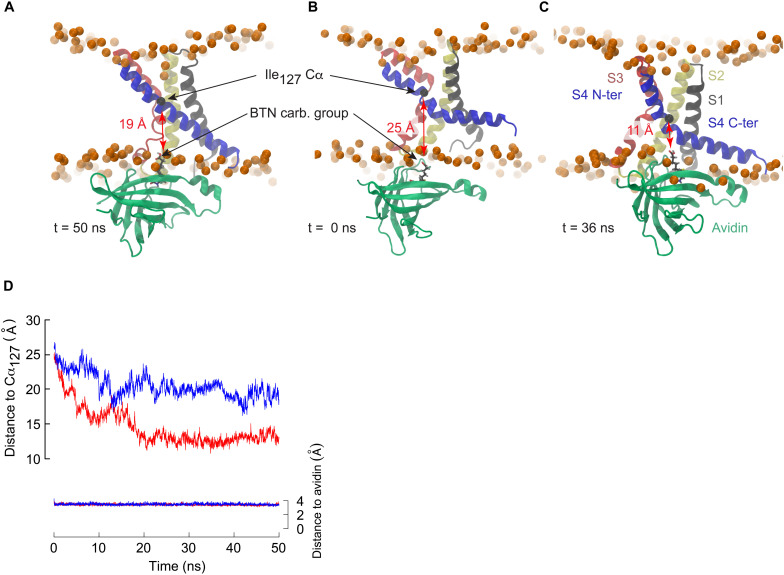
The breakage of S4 allows binding of avidin to biotinylated residues in the middle of S4. **(A–C)** Snapshots extracted from steered molecular dynamics simulations initiated with a VSD in which the S4 helix is straight (**A:** *t* = 50 ns) or kinked (**B:** *t* = 0 ns, **C:** *t* = 36 ns). Avidin is colored in green, and biotin is shown in stick representation with carbon black, oxygen red and hydrogen gray. The distance between biotin and the Cα (black sphere) of Ile_127_ is highlighted. **(D)** Time series of the distance between the carboxylic group of biotin and the Cα of Ile_127_ for the simulations with a straight S4 helix (blue) or a broken S4 helix (red). For control, the distance between the biotin and its binding site to avidin is shown (right *y* axis). Pulling forces were applied between the biotin and the Ile_127_ Cα, and between the biotin and the avidin binding site (see section “Materials and Methods”).

The MD simulations presented here show that the bending of S4 induced by membrane polarization reduces the distance required for avidin to bind to the biotinylated residues such that the S3a and S4 helices are not required to cross the membrane core. In order to determine how close an avidin molecule could come to residue Ile_127_ considering the conformational change of S4 observed under membrane hyperpolarization, we conducted a steered molecular dynamics (SMD) investigation involving the KvAP VSD and the monomeric avidin-biotin complex (Figure 5A). A moderate constant pulling force (see section “Materials and Methods”) was exerted between the biotin carboxylic acid functional group and the Cα of Ile_127_. A second pulling force was applied between the biotin and avidin to assure the stability of the complex. We performed the SMD simulations starting either from the X-ray structure or from a conformation harboring a broken S4, with an initial distance between the Cα of Ile_127_ and the biotin carboxylic acid of ∼25–26 Å. Whereas this distance stabilized to ∼19 Å during 50 ns of constant pulling in the case of a VSD with a straight S4 helix, it decreased steadily, attaining ∼ 11 Å, and stabilized at a value of ∼13 Å in the case of the broken S4 helix (Figure 5). This distance is significantly less than the length of the linker used experimentally, and thus the response of the VSD to membrane polarization is compatible with the accessibility experiments described above.

### How the Breakage of the S4 Helix Affects the Pore Domain

In order to test how the reorientation of the S4 C-ter segment might affect other α-helices and especially the pore domain, simulations were carried out based on the recently published structure of the whole channel, solved in the activated state (Tao and MacKinnon, 2019). Taking advantage of the available structure, molecular dynamics simulations of the whole channel embedded in a phospholipid bilayer were conducted to investigate what might be the initial response of the pore domain to the breakage of S4. The model described here involves the rupture of the Asp_62_-Arg_133_ salt bridge and the subsequent breakage of S4 at Gly_134_. In an initial simulation starting from the tetrameric activated state, Asp_62_ was protonated in order to facilitate its dissociation from Arg_133_ and Gly_134_ was pulled with a force constant of 120 kcal⋅mol^–1^⋅Å^–1^ toward the intracellular side of the membrane (see section “Materials and Methods”). The angle defined by the C-ter segment of S4 and the normal of the bilayer was monitored. A strong correlation (*R* = −0.93) was observed between the bending of S4 and the orientation of the S4 C-ter segment (the later allows a simpler description of the coupling between the VSD and the pore domain). Thus, in the following, the S4 α-helix is defined as broken if the S4 C-ter orientation is above 60 degrees and straight for values below 40 degrees. The simulation was stopped after 23 ns of pulling when one VSD harbored a broken S4 helix and other S4 helices also started to bend. An ensemble of 17 double bilayer systems based on the structure obtained after 23 ns of pulling were constructed (Asp_62_ was kept protonated) and exposed for an additional ∼ 240 ns to membrane potentials ranging from −0.8 to 0.0 V. Since each system contained two tetrameric channels, thus 8 VSDs, a total of 136 voltage-sensor domains were accessed.

The evolution through time of the root-mean-square deviation (RMSD) of the structures from the minimized cryo-EM structure (PDB accession code 6UWM) was monitored. As expected, the RMSD of the subunits increased steadily during the 23 ns of SMD. After release of the pulling force, the RMSD tended to stabilize at ∼ 2.5 Å in the systems under slightly polarizing conditions, whereas they continued to increase in the systems exposed to stronger membrane polarization (Supplementary Figure S3A). A statistical test was applied to reveal any relationship between the RMSD of various elements (S1, S2, S3 N-ter, S3 C-ter, S4 N-ter, S4 C-ter, S4, S5, S6, the pore helix, as well as the full-length channel) and the membrane potential. Except for S1, which showed a significant but weak response, only the full-length channel and the complete S4 helix showed strong and highly significant responses (Supplementary Figure S3B and Supplementary Data File bignucolo_berneche_dataset.xlsx). Interestingly, under a membrane potential of ∼0 V, the average RMSD of the S4 helices tended to decrease during the unrestrained simulations. Overall, this analysis suggests that the breakage of S4 was the main driver of the RMSD relationship with the membrane potential, whereas major conformational changes of other elements were not detected by the RMSD analysis.

**FIGURE 6:**
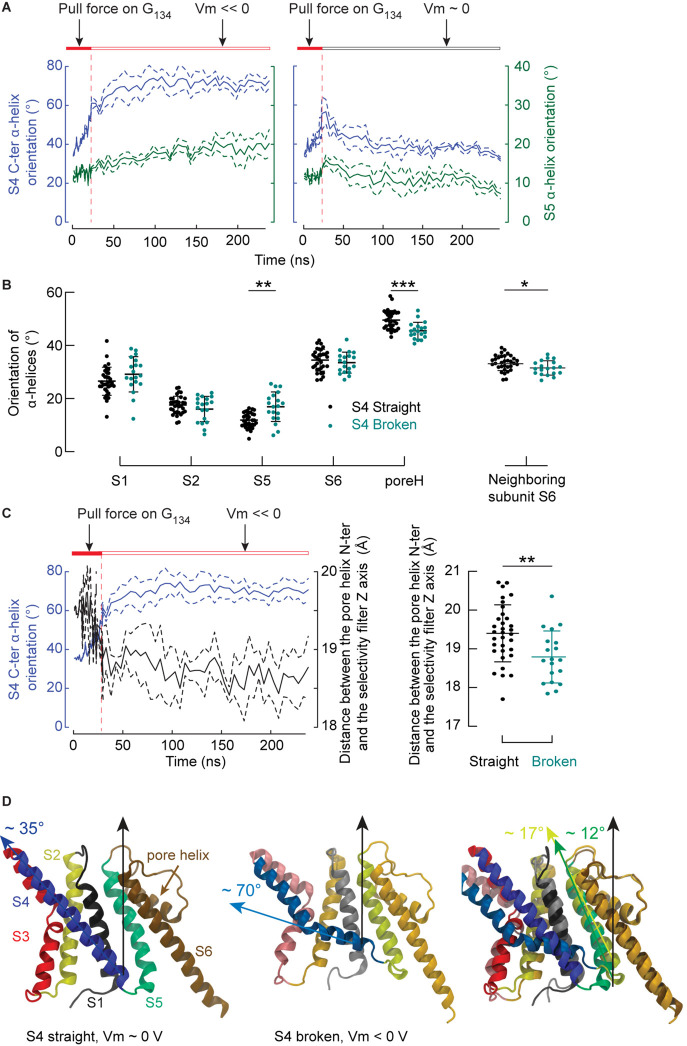
The breakage of S4 induces rearrangements affecting S5 and the pore helix. **(A)** Averaged time series of the orientation (means ± SEM) of the S4 C-ter segment (blue lines, left *y*-axis) and of S5 (dark turquoise, right *y*-axis). During the first 23 ns of simulations, the Cα atoms of G_134_ were pulled toward the intracellular space. The simulation was interrupted after 23 ns and trajectories involving double bilayer systems were conducted, in which the systems were either exposed to membrane hyperpolarization (left, *n* = 4) or depolarization (*n* = 3). The orientation of a helix was defined by the angle between the helix and the normal of the bilayer pointing toward the extra-cellular side. The horizontal bars summarize the tested conditions. **(B)** Orientations of the S1, S2, S5, S6 α helices, the pore helix and the S6 α helix of the neighboring subunit as a function of the S4 helix conformation (black: S4 straight, teal: S4 broken). **(C)** Left: Averaged time series of the orientation (means ± SEM) of the S4 C-ter segment (blue lines, left *y*-axis) and of the distance between the pore helix N-ter and the selectivity filter axis (black, right *y*-axis). Shown are simulations conducted under hyperpolarizing conditions. Right: Distances between the pore helix N-ter and the selectivity filter axis for individual subunits where S4 was straight (black) or broken (teal). **(D)** Representative molecular representations of a monomer in the activated state (left), after breakage of S4 under polarizing conditions (middle), and superposition of both structures after performing the structural alignment along S2, S6 and the selectivity filter. In the activated state, the helices are colored as follows: S1: gray, S2: yellow, S3: red, S4: blue, S5: dark turquoise, selectivity filter and S6: brown. Similar but more fade colors are used for the structures after S4 breakage. In all plots, the dots correspond to the mean of values extracted after discarding the first 180 ns of simulation for individual subunits and the means and standard deviations are shown. Stars indicate significant differences (*p < 0.05, **p < 0.01, ***p < 0.001) between ensembles following a single-way ANOVA corrected for multiple testing using the Šidák’s test.

We then focused on the response of S4 to the membrane potential and asked how this response might induce the closing of the pore. Under a membrane potential of ∼ 0 V, S4 tended to become straight again, confirming that the bent conformation of S4 is reversible. On the other hand, hyperpolarization increased the probability of observing S4 helices in a broken conformation with their C-ter segment oriented along the lipid head groups of the inner leaflet. These results are highly significant (*p* < 0.001), confirming that the mechanism observed on the isolated VSD occurs in the whole channel as well. Figure 6A shows illustrative trajectories where, after removing the pulling force, S4 either remains broken (at Vm << 0) or straightens back again (at Vm ∼ 0 V). This time series also suggests that the orientation of S5 is correlated with the orientation of the S4 C-ter segment. The orientation of all α helices (except S3, which is composed of two short segments) is shown as a function of the S4 C-ter orientation for all simulations (Figure 6B). Each subunit is represented by one point corresponding to the averaged orientation of a given helix. As shown in Figure 6B, the orientations of S5 and the pore helix, but not S1, S2 and S6, are correlated with that of the S4 C-ter α-helix. The angle between S5 and the normal to the bilayer is ∼ 11.8° in the activated state and ∼ 17.2° when the S4 C-ter α-helix slides along the lipid head groups (Figure 6D). Thus, this experiment shows that, when lying almost parallel to the membrane plane, the C-terminus of S4 pushes the intracellular extremity of S5 toward the symmetry axis of the pore domain, which results in a significant reorientation of S5 by ∼ 5–6 degrees.

Could this force exerted at the intracellular end of S5 initiate the closure of the pore? The extracellular end of S5 is sandwiched through hydrophobic contacts between S1 and the pore helix. Precisely, S5 shares essentially hydrophobic interactions with the N-ter of the pore helix (PH), e.g., interacting pairs of S5/PH residues: Ile_168_/Ala_186_, Phe_166_-Tyr_169_/Val_183_, Ala_165_/Ala_186_-Ala_190_. Since the upper part of S5 is restrained from the side by S1, which does not respond much to the conformational changes of S4, and its lower part is pushed by S4 toward the symmetry axis of the protein, the force is transmitted to the upper part of the pore helix. The pore helix also undergoes highly significant conformational changes, which are correlated with the breakage of S4. The angle between the pore helix and the normal to the bilayer is ∼ 49° when S4 is straight and ∼45° when S4 is broken (p < 0.001).

Further analysis shows that the lateral distance from the pore helix N-ter to the symmetry axis of the channel correlates with the state of S4. Figure 6C reports the time evolution of this distance for the same sub-sample of simulations as in Figure 6A, and indicates a correlation between the bending of S4 and the pore helix N-ter distance to the channel symmetry axis, which is further confirmed when performing the statistical analysis on all the simulations (p = 0.004, Figure 6C, right). The same analysis showed a weak probability for the center of the pore helix (p ∼ 0.09) and no relation for the C-ter of the pore helix (data not shown). The conclusion of these three analyses is that the center of rotation of the pore helix lies at its C-ter. Thus, the force exerted by the S4 C-ter is transmitted to the upper part of the pore helix via S5. The pore helix rotates around its C-ter, so that its N-ter moves toward the symmetry axis of the protein, which could constrict the pore and potentially lead to the closure of the selectivity filter.

**FIGURE 7:**
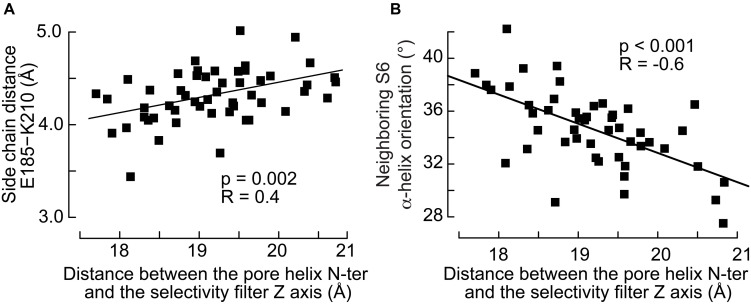
The inward movement of the pore helix affects its interactions with the neighboring S6 helix. **(A)** Distance between the side chain of Glu_185_ in the pore helix and Lys_210_ in the neighboring S6 helix as a function of the distance between the pore helix N-ter and the selectivity filter axis. **(B)** Orientation, defined by the angle between the helix and the normal of the bilayer pointing toward the extra-cellular side, of the neighboring S6 helix as a function of the distance between the pore helix N-ter and the selectivity filter axis. The *p*-values of linear regressions and the coefficients of correlation are indicated.

The movement of the pore helix was further shown to modulate the interactions between subunits. The simulations suggest that the reorientation of the pore helix and the radial translation of its N-ter toward the pore correlate with the distance between Glu_185_ in the pore helix and Lys_210_ located in the upper part of the S6 helix of the neighboring subunit (Figure 7A): the side chains of the two residues are closer to each other when the N-ter of the pore helix is closer to the protein symmetry axis. These observations raise the hypothesis that the modulation of a salt bridge between these residues may contribute to the state transitions of the channel. The analysis presented above showed no correlation between the S4 conformation and S6 of the same subunit. However, we find that the movement of the pore helix affects significantly the orientation of the closest neighboring S6 helix (Figure 7B). The angle between the S6 helix and the normal to the bilayer lies at 35–40° when the pore helix of the neighboring subunit is far from the symmetry axis and 30–35° when it is nearer. On the other hand, the orientation of S6 is 36° resp. 34° when S4 of the neighboring subunit is straight resp. broken, with a weaker correlation than seen for the S5 and pore helices (Figure 6B).

### The Sequence Features of the KvAP S4 Helix Are Specific to Prokaryotes

The kink in S4 occurs at the level of a Gly residue (Gly_134_) located eight positions downstream from the four signature voltage-sensing basic residues (Arg_117_, Arg_120_, Arg_123_ and Arg_126_). Several prokaryotic potassium channels display a sequence that meet this feature (Supplementary Figure S4). However, the most resembling eukaryotic sequences contained a much shorter segment, generally 3 residues, between the signature basic residues and the next Gly. Despite some structural similarities between KvAP and HCN1 (non-swapped topology, long S4 helix, breakage of S4 under hyperpolarization), the sequences of the S4 N-ter helices differ significantly.

## Discussion

This study reveals a novel response of KvAP to cell polarization that consists in the concurrent disruption of the salt bridge between Asp_62_ in S2 and Arg_133_ in S4 and the breakage of S4 at the level of Gly_134_. Whereas the rupture of the Asp_62_-Arg_133_ salt bridge has been reported as a response to polarization (Freites et al., 2012), the current study shows that the rupture of the salt bridge induces the breakage of S4. These conformational changes allow for the translation of the upper part of S4 along its principal axis and toward the intracellular side of the bilayer. This implies that a tethered biotin on the external half of S4 is accessible to avidin from the intracellular compartment, bringing a biophysically coherent explanation to the accessibility experiments described by Jiang et al. (2003b) and Ruta et al. (2005).

Sequence alignment reveals that, while the specific sequence of the KvAP is hardly found in any eukaryotic voltage-gated potassium channels (Supplementary Figure S4), it is present in several archaea and prokaryotes, among which many pathogens, making it a potential target for antibiotic investigations. Many of the prokaryotic voltage dependent K channels with a S4 sequence reminiscent of KvAP belong to the anaerobic Bacteroides (see Supplementary Figure S4), which are of significant clinical relevance. *Bacteroides fragilis* infections display a mortality of 20%, which rises to more than 60% if left untreated (Wexler, 2007). The incidence of anaerobic bacteraemia is relatively low (less than 3%), but the associated mortality is above 20% (Kim et al., 2016). In the same line, *Bacteroides pyogenes* causes life-threatening human wound infections (Lau et al., 2016; Park et al., 2016), and *Bacteroides thetaitoamicron*, which can exacerbate *E. coli* and *Clostridium difficile* infections, is the second most common infectious anaerobic gram-negative bacteria (Curtis et al., 2014). Mutation experiments involving the *E. Coli* Kch potassium channel suggested that it maintains the membrane potential and could prove essential under certain stress conditions, like higher external potassium concentration (Kuo et al., 2003). The relevance of potassium channels as potential antibacterial targets is elegantly demonstrated by the observation that gastric colonization by *Helicobacter pylori* is impaired when lacking its potassium channel HpKchA (Stingl et al., 2007). The WHO listed antimicrobial-resistance as one of the biggest threats to humanity. Thus, the prokaryotic specific mechanism identified in the current study may be exploited as a selective target for several deadly pathogens, some of them harboring multiple antibiotic resistance (β-lactam, Carbapenems and other antibiotics) (Wang et al., 2000; Teng et al., 2004; Fernández-Canigia et al., 2012; Hartmeyer et al., 2012).

Interestingly, experimental (Chakrapani et al., 2008; Butterwick and MacKinnon, 2010; Shenkarev et al., 2010) and computational (Sands et al., 2006) investigations have reported either that the S4 of KvAP may bend near the middle of the bilayer, or that the Asp_62_-Arg_133_ salt bridge may break (Freites and Tobias, 2015). In an NMR structure determination of the KvAP VSD, a loss of helical periodicity was identified at the level of Gly_134_, suggesting that the S4 helix might be constituted of two helices connected by a hinge comprising Ile_131_, Ser_132_ and Arg_133_ (Shenkarev et al., 2010). Intriguingly, three of the 20 conformations deposited for the KvAP VSD NMR solution structure (code 2KYH) display a slightly bended S4 (Butterwick and MacKinnon, 2010). Whereas experimental or computational studies support the idea of a hinge in the middle of S4 or the rupture of the salt bridge, the present study shows for the first time that these two conformational changes happen simultaneously upon polarization and that they lead to the charge transport observed experimentally and generally interpreted as gating current.

In a recent cryo-EM study on the HCN channel, a hyperpolarization activated potassium channel, a disulfide bridge was generated between F186C in helix S2 and S264C in helix S4 with the aim of mimicking hyperpolarized conditions. Consequently, the VSD was trapped in a presumably activated state, characterized by a S4 α helix broken at the level of the disulfide bridge and a sliding movement of the external part of S4 toward the intracellular side (Lee and MacKinnon, 2019). One key feature of KvAP is that S4 is 13 residues longer than the corresponding helix in Kv1.2 and Kv1.2/Kv2.1. The S4 helix of the HCN channel is of similar length and its breakage was shown to occur upon membrane hyperpolarization (Kasimova et al., 2019). The breakage of the S4 helix into two smaller helices was thus suggested to be essential to hyperpolarization gating. Our unrestrained simulations reveal that this feature is not unique to channels activated by membrane hyperpolarization, but is also observed in a channel activated by depolarization, like KvAP. This model remains consistent with the hypothesis that the breakage of S4 is a general feature of non-swapped ion channels, as are HCN and KvAP. The functional distinction between the two families of channels, either activated by hyperpolarization like HCN or depolarization like KvAP, could reside in the way their VSD is coupled to the pore domain.

**FIGURE 8:**
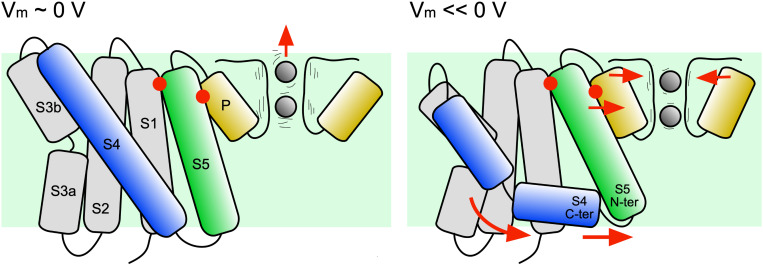
Mechanistic model of the first steps of the transition from the activated to the resting state. The hypothetic model is shown with the channel under depolarized **(left)** and hyperpolarized **(right)** conditions. The movements deduced from the MD trajectory analyses are indicated by red arrows. After the hyperpolarization induced breakage of S4, the S4 C-ter segment re-orients parallel to the membrane plane. The S4 N-ter exerts a translational movement toward the intracellular side. Steered by the S4 C-ter, the N-ter of S5 is displaced. S5 C-ter is, however sandwiched between S1 and the pore helix (red dots). Due to this constraint, a part of the movement of S5 is transmitted to the pore helix, which is pushed toward the symmetry axis of the channel, inducing a constriction of the selectivity filter. This constriction may reduce the fluctuations of the carbonyl groups lying the pore and ion permeation.

Taking advantage of the recently solved whole channel structure of KvAP, the initial responses of the pore domain to membrane polarization and subsequent breakage of S4 was investigated. The simulations show that when the S4 C-ter segment slides along the head groups of the intracellular side of the membrane, the S5 α-helix reorients with its intracellular terminus moving toward the symmetry axis of the channel (see Figure 8). On the extracellular side, the S5 C-ter shares several hydrophobic interactions with the pore helix, which at its turn is also driven in an inward radial movement toward the selectivity filter. It results in a strong correlation between the state of helix S4 and the orientation of the pore helix, suggesting that, upon hyperpolarization, the pore helix N-ter would exert a constrictive force on the selectivity filter. Correlated with the reorientation of the pore helix is the reorientation of the neighboring S6 helix and the stabilization of a salt bridge connecting the pore helix and the neighboring S6 helix. Though S6 is not seen occluding the pore, it remains possible that such conformational change could take place at a later stage of the transition.

The involvement of S5 is appealing as it was shown that in the pH gated KcsA channel it is mainly the outer helix of the pore domain, corresponding to S5, that transmits the gating signal to the selectivity filter where activation gating actually takes place (Heer et al., 2017). This model suggests that constriction of the selectivity filter by the pore helix reduces the fluctuations of the carbonyl groups lying the pore and coordinating the permeating ions to a point that ions can no longer diffuse. It implies that in the resting state the selectivity filter exerts a high ion binding affinity, which traps the ions and prevents permeation. Mutagenesis data suggest that a similar mechanism could apply to voltage-dependent K channels (Lees-Miller et al., 2009; Garneau et al., 2014). Such reduction of the selectivity filter fluctuations was not detected in our simulations. Nevertheless, our investigations revealed how the conformational changes of S4 impact on the conformation of residues in close vicinity of the selectivity filter.

## Conclusion and Contribution to the Field

We show that the response of the KvAP voltage-sensor domain to membrane polarization consists in two co-occurring conformational changes: the rupture of the Asp_62_-Arg_133_ salt-bridge and the breakage of the S4 helix. This finding is consistent with the hypothesis that the breakage of S4 is a general feature of non-swapped Kv channels. Upon polarization, the N-ter segment of the S4 helix translates toward the intracellular side of the membrane, which allows to solve previously paradoxical avidin accessibility measurements. Simulations involving the recently solved whole structure of KvAP show how these conformational changes induce a reorientation of the S5 and pore helices, putatively leading to the constriction and closure of the selectivity filter. This study also links mechanistic insights in prokaryotic potassium channel membrane potential sensing with the perspective of finding new antibiotic targets.

## Data Availability Statement

The datasets of this study are provided as bignucolo_berneche_dataset.xlsx in the Supplementary Data.

## Author Contributions

OB and SB conceived the project and wrote the manuscript. OB carried out the computational work. Both authors contributed to the article and approved the submitted version.

## Conflict of Interest

The authors declare that the research was conducted in the absence of any commercial or financial relationships that could be construed as a potential conflict of interest.

## Abbreviations

COM: center of mass
MD: molecular dynamics
SMD: steered molecular dynamics
Vm: membrane electrostatic potential
VSD: voltage-sensor domain.
