# Supplementary material for: The Voltage-Dependent Deactivation of the KvAP Channel Involves the Breakage of Its S4 Helix
Source: Front Mol Biosci. 2020 Jul 29;7:162. doi: 10.3389/fmolb.2020.00162 (PMC7403406; doi:10.3389/fmolb.2020.00162)
Supplement: Supplementary file 2 [file Data_Sheet_1.pdf]

Supplementary Material

The voltage-dependent deactivation of the KvAP channel involves the breakage of its S4 helix

Olivier Bignucolo, Simon Bernèche

Preprint

A previous version of this manuscript was submitted to bioRxiv and can be found at:
doi: https://doi.org/10.1101/2019.12.28.889881
Note, however, that this preprint was produced before the publication of the full-length cryo-EM structure of KvAP. Therefore, the computational data involving the whole channel, which show how the breakage of S4 affects the pore domain, are not included in the preprint version.

1. Ion and water transport

In a previous molecular dynamics study, Freites et al. reported the formation of a water pore through the KvAP VSD, pointing the Asp_{62}-Arg_{133} salt bridge rupture as a requirement for water transport (Freites, Tobias et al. 2006). In two simulations involving the isolated VSD, we observed a potassium ion moving from the extracellular to the intracellular compartment together with several water molecules. However, we propose that this opening is rather transient, and that further sliding of S4, though not observed in our rather short simulations, would allow Arg_{133} to form new interactions with acidic residues located more toward the intracellular side, namely Asp_{72} in S2 and Glu_{93} in S3. These new interactions would then restrict the transient opening of a pore in the VSD.

2. The interactions with the membrane

We noticed a narrowing of the lipid bilayer in the vicinity of the protein. Even residues Arg_{123} and Arg_{126}, which are located towards the middle of the bilayer, formed hydrogen bonds with the lipids head groups. The water filled cavities, on both sides of the membrane, also induced rearrangements of the phospholipids. As shown in Figure 3B, a few phosphate groups entered in the water filled cavity, although they generally remained at the most external part of it. We wondered whether the formation of the kink, which may further enlarge the water filled cavity on the intracellular side, would affect the structure of the bilayer. As shown in Supplementary Figure 1, the intracellular leaflet thickness was ~ 20 Å resp. 17 Å for lipids situated far from resp. near the VSD. However, the thinning of the bilayer was the same whether S4 formed a kink or not. To eliminate any doubts, we further plotted the thickness of the membrane as a function of the bending of S4. We also analyzed the thickness of the lower leaflet as a function of the 'height' of the last residue of S4, defined as the distance along the normal to the bilayer between the Cα atom of Leu_{148} and the average 'height' of the C316 atoms of the lipids, representing the center of the bilayer. In both analyzes (data not shown), there was no relationship between the thickness of the leaflet and the conformation of S4, which demonstrates that

the kink did not affect the penetration of the lipid head groups into the membrane. On the other hand, the polar head groups tend to reorient in the vicinity of a transmembrane protein. We then wondered whether the formation of the kink in S4 would modify the polar head orientation. The orientation of the phosphocholine head groups is defined by the angle between the Phosphorus to Nitrogen vector and the normal to the bilayer. The average value of this angle was ~ 71 degrees resp. ~ 64 for lipids situated far from resp. near the VSD. The kink in S4, however, had no impact on the reorientation of the lipid polar heads. It is thus concluded that whereas the membrane structure was modified in the vicinity of the channel, these rearrangements were not affected by the disruption of the Asp_{62}-Arg_{133} salt bridge and the kink of S4.

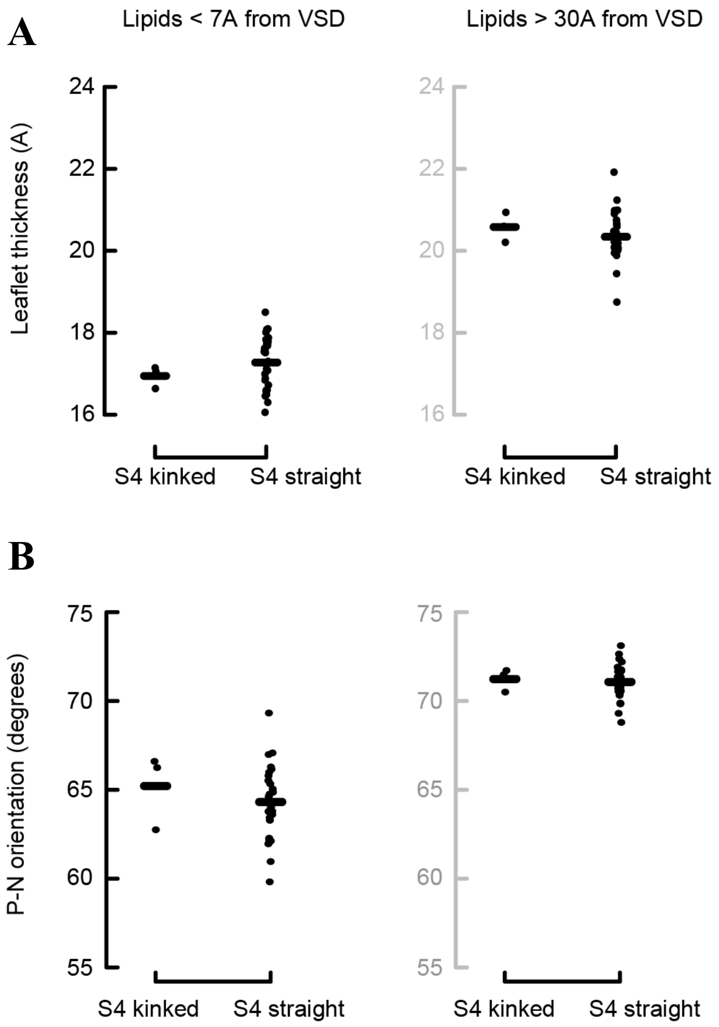

Figure S1. The local membrane deformation is independent of the kink in S4. (A) Thickness of the lower leaflet of the membrane for lipids situated close (< 7 Å) resp. far (> 30 Å) from the VSD during the last 20 ns of the trajectories. The data are further split in trajectories exhibiting a kink in S4 or not. **(B)** Orientation of the phosphocholine head groups of the lipids, data subdivision as in A. The head group orientation was defined as the angle between the P-N vector and the normal to the bilayer, where P and N stand for the phosphorous and nitrogen atoms.

3. Determination of the simulation time required to observe decorrelated S4 conformations

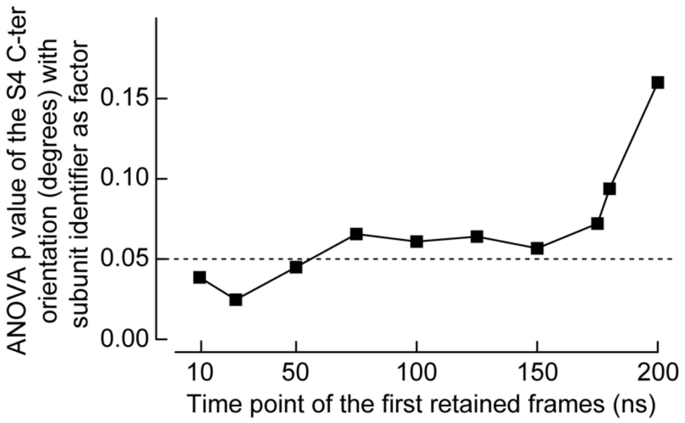

Figure S2. Decorrelation from the initial conditions

Correlation, expressed as the p value of a single-way ANOVA, of the S4 C-ter orientation as a function of the initial length of simulation discarded for analysis. The test accessed the orientation of the S4 C-ter with respect to the normal of the bilayer and took the subunit identifier as a fixed factor. See the Method section 2.3 for a complete explanation.

4. RMSD Analysis

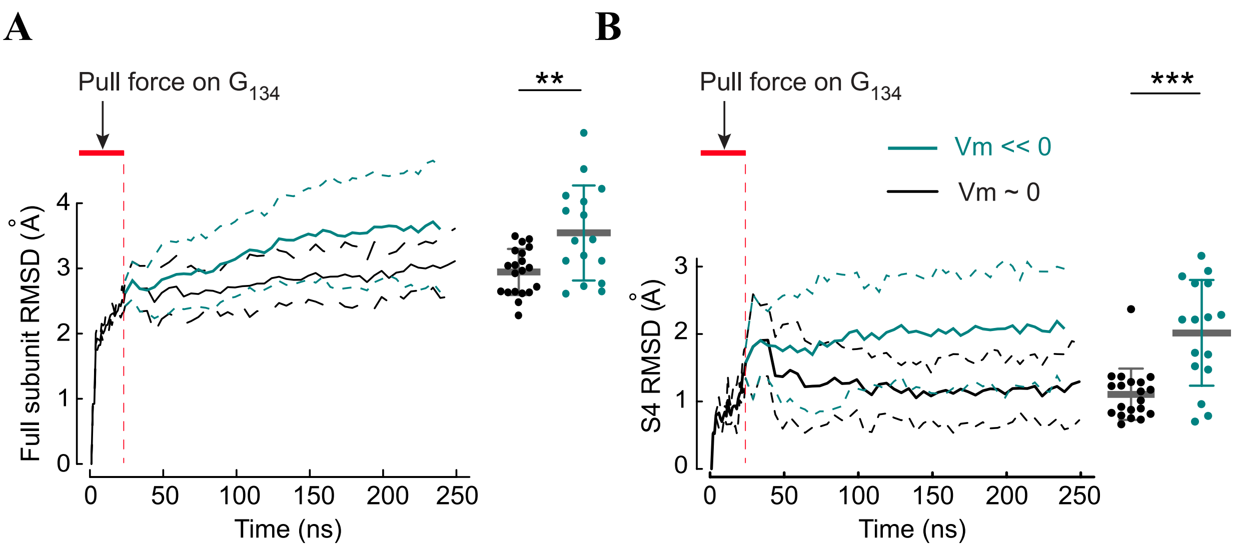

Figure S3. The RMSD from the initial structure depends on the membrane potential and the S4 conformational switch is the main driver. Time evolution of the subunits **(A)** and S4 **(B)** RMSD from the cryo-EM structure. The data are further split into simulations conducted under a membrane potential > -0.4 V (black symbols, 5 trajectories) or < -0.65 V (teal, 4 trajectories). The quantification reports the averages, errors and individual values after removing of the first 180 ns of unrestrained simulation, corresponding to t ≥ 203 ns on the figure.

5. The sequence features of the KvAP channel are specific to prokaryotes

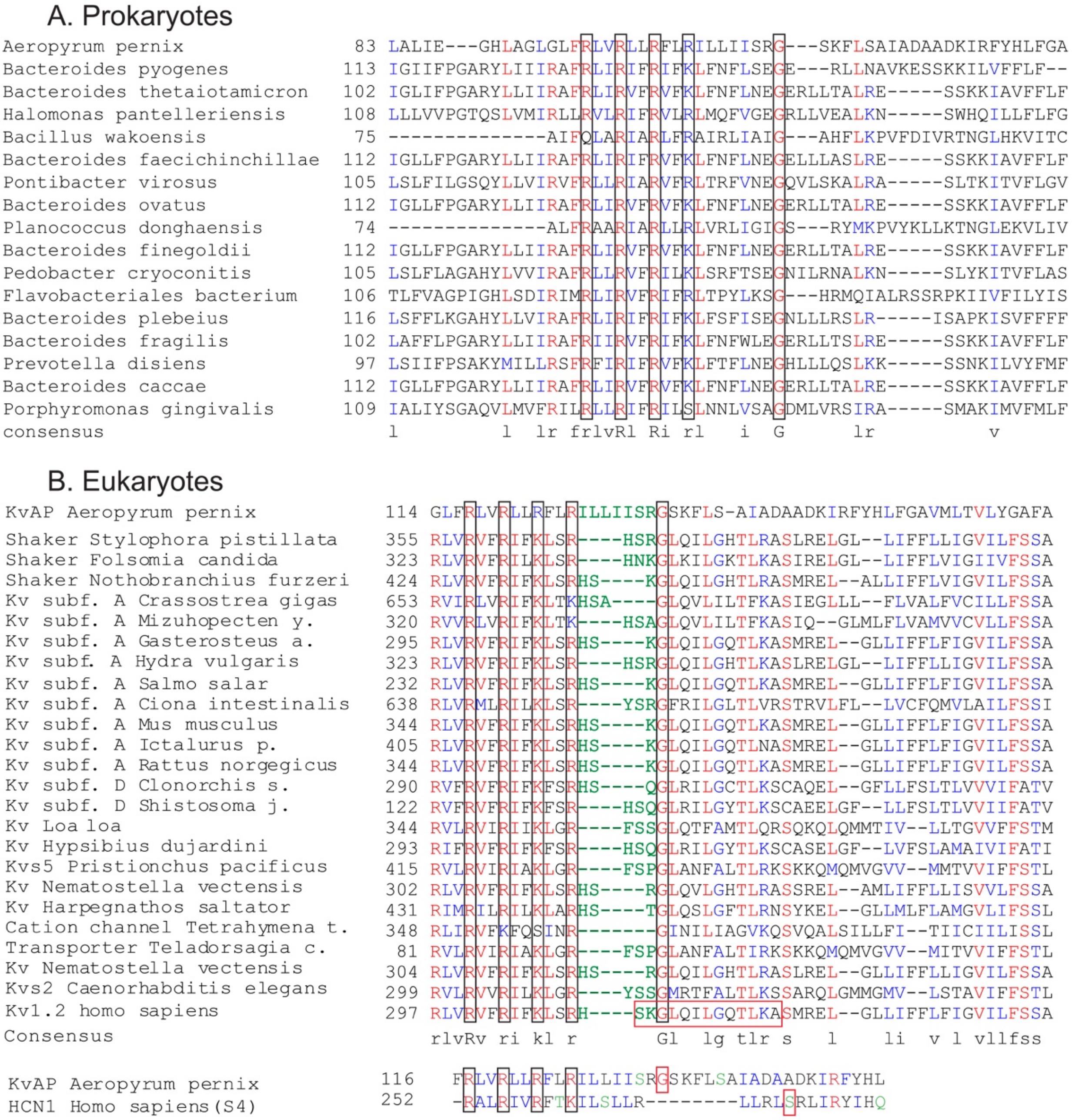

Figure S4. The sequence features of the KvAP S4 helix are specific to prokaryotes. The figure shows sequence alignment of a subset of KvAP S4 containing the four voltage sensing basic residues with the best hits found among **(A)** prokaryotes or **(B)** eukaryotes. The alignments display from left to right, the name of the channel or the family it belongs to, the species, the number of the first residue listed and the sequence colored as a function of the conservation. The consensus is displayed at the bottom when appropriate. In **(B)**, the region highlighted in green

shows that no eukaryotic sequence was found that could be aligned with KvAP without gaps between the signature Arginine residues and the Gly_{134}. The residues contoured in red – human Kv1.2 – form the S4-S5 linker in the Kv1.2 X-ray structure (PDB accession code 3LUT). In the alignment of the KvAP and HCN1 channels (bottom), the residues at which the breakage of the S4 helices was observed (KvAP, GLY_{134} and HCN1, SER_{173}) are contoured in red.

References

Freites, J. A., D. J. Tobias and S. H. White (2006). "A voltage-sensor water pore." Biophys J **91**(11): L90-92.

Hurlbert, S. H. (1984). "Pseudoreplication and the Design of Ecological Field Experiments." Ecological Monographs **54**(2): 187-211.
